# Draft genome sequence of *Pseudomonas protegens* M4 and *Citrobacter braakii* M34 isolated from the sunflower endosphere

**DOI:** 10.1128/mra.01296-25

**Published:** 2026-03-10

**Authors:** Olubukola Oluranti Babalola, Muritala Muhammed, Ayansina Segun Ayangbenro

**Affiliations:** 1 Food Security and Safety Focus Area, Faculty of Natural and Agricultural Sciences, North-West University 274466 , Mmabatho, South Africa; 2 Department of Life Sciences, Imperial College London 98991 , Ascot, Berkshire, United Kingdom; Rochester Institute of Technology, Rochester, New York, USA

**Keywords:** genome sequencing, plant growth promotion, secondary metabolites, annotation

## Abstract

This study presents the draft genome sequences of two bacterial strains, *Pseudomonas protegens* M4 and *Citrobacter braakii* M34, isolated from the roots of *Helianthus annuus* (sunflower). Genome assembly yielded 6,900,000 bp for M4 with an *N*_50_ of 810,200 bp and 4,900,000 bp for M34 with an *N*_50_ of 2,700,000 bp.

## ANNOUNCEMENT

Endophytic bacteria, particularly members of the Proteobacteria, enhance plant growth and stress tolerance by colonizing internal plant tissues without causing harm and promoting plant vigor through phytohormone production, nutrient acquisition, pathogen suppression, and stress adaptation, as demonstrated by recent meta-omics studies (1–3). Despite their importance, genomic data on sunflower-associated endophytes remain limited. Here, we report the draft genome sequences of *Pseudomonas protegens* M4 and *Citrobacter braakii* M34 isolated from sunflower roots. Sunflower root samples were collected in July 2024 from Delareyville, North West Province, South Africa (27.11828° S, 25.88914° E). The cultivar used was PAN 7080 (PANNAR seeds, batch no. 5450598). Roots were surface-sterilized and serially diluted, and 0.1-mL aliquots of the 10⁻³ dilution were plated using the pour plate method on *Pseudomonas* isolation agar (PIA; Scharlab S.L., Spain) and King’s B agar (Oxoid, UK) following established procedures (4). Plates were incubated at 28°C for 24 h, and distinct colonies were purified by repeated subculturing. Genomic DNA was extracted from pure cultures grown on nutrient agar (Oxoid, UK) at 28°C for 24 h using the Zymo Quick-DNA Miniprep Kit (Zymo Research, USA) according to the manufacturer’s protocol. DNA quality and concentration were assessed using a NanoDrop spectrophotometer (Thermo Fisher Scientific, USA). Genome sequencing was performed by Novogene Co., Ltd., (Singapore) using the Illumina Novaseq X Plus platform. Libraries were prepared using an Illumina Nextera DNA Flex Kit and assessed using an Agilent fragment analyzer (Agilent Technologies, USA) and Qubit fluorometry. Preliminary taxonomic identification was performed using 16S rRNA gene sequencing and National Center for Biotechnology Information (NCBI) BLAST analysis. Genome analysis was performed in KBase (5). Read quality was assessed with FastQC v0.12.1 (6), and low-quality bases and adapters were removed using Trimmomatic v0.39 (7) using SlidingWindow:4:20, Leading:3, Trailing:3, and MinLen:50. After trimming, 8,813,994 and 8,213,592 high-quality paired-end reads were retained for *P. protegens* M4 and *C. braakii* M34, respectively. Estimated sequencing coverage was ~192× for M4 and ~251× for M34 calculated from total trimmed bases and estimated genome sizes. Genome assembly was performed using SPAdes v3.15.3 (8) with default settings. Genome completeness and contamination were assessed with CheckM v1.0.18 (9), yielding 99.68% completeness and 1.05% contamination for M4 and 99.82% completeness and 2.94% contamination for M34. Annotation was conducted using the NCBI Prokaryotic Genome Annotation Pipeline (PGAP) (10). The presence of secondary metabolites, such as arylpolyenes, non-ribosomal peptide synthetases, and terpenes, was revealed in both strains with antiSMASH v8.0.2 (11). Default parameters were used, unless otherwise specified. *P. protegens* M4 genome comprised 13 contigs totaling ~6,900,000 bp with a guanine and cytosine (GC) content of 63.5% and an *N*_50_ of 810,200 bp. The *C. braakii* M34 genome comprised 12 contigs totaling ~4,900,000 bp with a GC content of 52.0% and an *N_50_* of 2,700,000 bp. PGAP analysis identified genes, including those associated with phosphate and nitrogen metabolism, stress tolerance, and exopolysaccharide biosynthesis. Assembly statistics are summarized in Table 1.

**TABLE 1:** Whole genome properties of *Pseudomonas pretegens* M4 and *Citrobacter braakii* M34

Genomic parameters	*Pseudomonas pretegens* M4	*Citrobacter braakii* M34
Reads after trimming	8,813,994	8,213,592
Genomic size (bp)	6,900,000	4,900,000
Total ungapped length (bp)	6,900,000	4,900,000
Number of contigs	13	12
GC contents (%)	63.5	52.0
Genome coverage (×)	192	251
*N*_50_ value (bp)	810,200	2,700,000
*L*_50_ value (bp)	4	1
tRNA (count)	51	77
rRNA (count)	1	3
Total number of genes	6,237	4,740
Number of coding genes	6,112	4,571
Pseudo genes	68	78
CDSs	6,810	4,649
CheckM completeness (%)	99.68%	99.82%
CheckM contamination (%)	1.05%	2.94%
GenBank accession number	JBQRVA000000000	JBQUYN000000000
SRA accession number	SRR35264040	SRR35264041

## Data Availability

*Pseudomonas protegens* strain M4 and *Citrobacter braakii* M34 whole-genome projects have been deposited at DDBJ/ENA/GenBank under accession numbers JBQRVA000000000 (https://www.ncbi.nlm.nih.gov/datasets/genome/GCF_052371255) and JBQUYN000000000 (https://www.ncbi.nlm.nih.gov/datasets/genome/GCF_052409275), respectively. The versions described in this paper are JBQRVA010000000 and JBQUYN010000000, with the raw reads available under BioSample accession numbers SAMN50614363 and SAMN50614364 under the same Bioproject accession number PRJNA1305676. The strains have sequence reads archived under accession numbers SRR35264040 and SRR35264041, respectively. The complete antiSMASH biosynthetic gene cluster predictions generated for this study are publicly available on Figshare via https://doi.org/10.6084/m9.figshare.30207388.
